# Error in Byline

**DOI:** 10.1001/jamanetworkopen.2026.41571

**Published:** 2026-10-02

In the Original Investigation titled “A Multilevel Intervention to Identify Individuals for Genetic Testing and Treatment: The IGNITE-TX Pilot Randomized Clinical Trial,” published August 27, 2026, Melissa K. Frey’s middle initial was omitted from the byline. This article has been corrected.^1^
